# Human West Nile Virus Disease Outbreak in Pakistan, 2015–2016

**DOI:** 10.3389/fpubh.2018.00020

**Published:** 2018-02-27

**Authors:** Erum Khan, Kelli L. Barr, Joveria Qais Farooqi, Dhani Prakoso, Alizeh Abbas, Zain Yar Khan, Shanze Ashi, Kehkashan Imtiaz, Z. Aziz, Faisal Malik, John A. Lednicky, Maureen T. Long

**Affiliations:** ^1^Department of Pathology and Laboratory Medicine, Aga Khan University, Karachi, Pakistan; ^2^Department of Comparative Diagnostic and Population Medicine, College of Veterinary Medicine, University of Florida, Gainesville, FL, United States; ^3^Department of Environmental and Global Health, Emerging Pathogens Institute, University of Florida, Gainesville, FL, United States

**Keywords:** West Nile virus, Dengue virus, Japanese encephalitis virus, encephalitis, arboviral disease

## Abstract

Like most of the world, Pakistan has seen an increase in mosquito-transmitted diseases in recent years. The magnitude and distribution of these diseases are poorly understood as Pakistan does not have a nation-wide system for reporting disease. A cross-sectional study to determine which flaviviruses were causing of arboviral disease in Pakistan was instituted. West Nile virus (WNV) is a cause of seasonal fever with neurotropic findings in countries that share borders with Pakistan. Here, we describe the active and persistent circulation of WNV in humans in the southern region of Pakistan. This is the first report of WNV causing neurological disease in human patients in this country. Of 997 enrolled patients presenting with clinical features suggestive of arboviral disease, 105 were positive for WNV IgM antibodies, and 71 of these patients possessed WNV-specific neutralizing antibodies. Cross-reactivity of WNV IgM antibodies with Japanese encephalitis virus (JEV) occurred in 75 of these 105 patients. WNV co-infections with Dengue viruses were not a contributing factor for the severity of disease. Nor did prior exposure to dengue virus contribute to incidence of neurological involvement in WNV-infected patients. Patients with WNV infections were more likely to present with altered mental status, seizures, and reduced Glasgow Coma scores when compared with JEV-infected patients. Human WNV cases and vector numbers exhibited a temporal correlation with climate.

## Introduction

West Nile virus (WNV) is a single-stranded, enveloped positive-sense RNA flavivirus that is maintained in nature by an enzootic cycle between birds and blood-feeding *Culex* mosquitoes (family *Culicidae*) (1). *Culex* spp. are widely distributed over the world and have been reported to feed on birds, humans, horses, and other mammals (2). Outbreaks of WNV disease have been reported in Europe, America, Africa, and parts of Asia (3–5). Serological evidence in humans and vector competence for WNV in Pakistan has been reported as early as 1982 (6, 7). In recent years, WNV has been identified in horses and humans in Pakistan and its neighboring countries, Iran, Afghanistan, and India as a cause of human encephalitic disease (8–13). Surveillance studies in these surrounding countries report WNV circulation in several *Culex* species as well as several wild, migratory, and domestic birds and water fowl (14–19).

With the dissolution of the Pakistan Federal Ministry of Health in 2011, reporting and surveillance for arboviral diseases have become largely non-existent and, when performed, is highly localized and mostly funded by foreign granting agencies. Pakistan is at a continual risk of suffering large epidemics of vector-borne diseases for a variety of reasons. Geopolitical instability, annual flooding, and poor health care infrastructure lead to frequent internal human and animal displacement. Moreover, the construction of super highways and shipping ports for efficient travel between Africa and China has left Pakistan vulnerable to the introduction of foreign diseases. Pakistan has recently witnessed an increased burden of arboviral infections, primarily from Crimean-Congo hemorrhagic fever virus, WNV, Chikungunya virus (CHIKV), and Dengue viruses (DENVs) affecting the economic, social, and political aspects of people’s lives (7, 20–22). More recently, active circulation of DENV, WNV, and CHIKV has been reported in humans living in the Punjab province of Pakistan (22).

Pakistan lacks a holistic surveillance system for arbovirus infections; therefore, there are limited data regarding types of vectors circulating, their temporal trends, and their correlations with climatic factors and human/animal cases. In light of the heightened alert of epidemic outbreaks and the global spread of arboviruses like WNV, CHIKV, and Zika virus, active surveillance for arboviruses in Pakistan is desperately needed for effective implementation of preventive and control strategies. Here, we present results of a multiyear seroprevalence and mosquito surveillance project initiated in 2015 that analyzed human arboviral exposure, mosquito vectors, and their correlation with climatic factors.

## Materials and Methods

### Patient Enrollment

**Figure 1:**
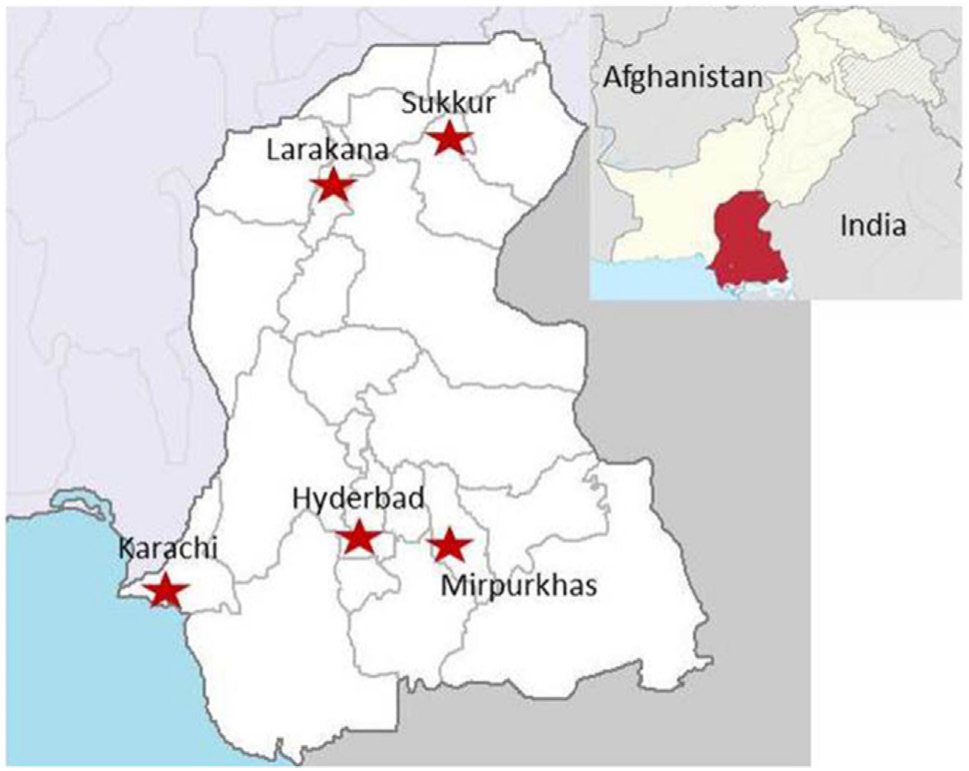
Locations of the patient enrollment sites in the Sindh region of Pakistan (8). Sites included medical colleges, teaching hospitals, and civil hospitals.

A cross-sectional, observational study was performed to identify which arboviruses [DENV, WNV, and Japanese encephalitis virus (JEV)] were the cause of acute undifferentiated febrile illness in selected basic health units and/or district hospitals of the Sindh region of Pakistan (8). Patients were enrolled under informed consent procedures, which were reviewed and approved by the Ethics Review Committee at Aga Khan University (3183-PAT-ERC-14) and the Institutional Review Board at the University of Florida (201500908). All enrolled subjects gave written informed consent in accordance with the Declaration of Helsinki. Patients presenting with the CDC clinical description of arboviral disease including findings of rash, headache, arthralgia, myalgia, gastrointestinal distress, acute hemorrhagic fever, acute flaccid paralysis, encephalitis, meningitis, and/or unexplained fever were recruited (23). Patients younger than 10 years and older than 90 years were excluded. Briefly, all patients were tested for DENV NS1 antigen unless affected primarily by neurologic abnormalities. All NS1-negative sera were evaluated for exposure to WNV or JEV *via* IgM capture ELISA for JEV and WNV. Five study sites were established, and personnel were trained throughout the Sindh province in Pakistan (Figure 1) (8). These sites included medical colleges, teaching hospitals, and civil hospitals.

### Diagnostic Criteria

Patients were confirmed positive for virus exposure according to CDC criteria, which requires “virus-specific IgM antibodies in serum with confirmatory virus-specific neutralizing antibodies in the same or a later specimen” (23). Patients were classified as a suspect case if they had virus-specific IgM antibodies in serum but with a neutralizing titer of less than 50% at the highest concentration of serum. For cross-reactive specimens, patients were confirmed positive when they had virus-specific IgM antibodies and neutralizing antibodies in the same specimen that exhibited the highest level of neutralization for one of the viruses in question. Cross-reactive patients were classified as suspect according to the greatest amount of virus-specific IgM antibodies. Patients were considered positive for DENV co-infection if they had DENV-specific NS1 antigen. Patients were considered as having prior DENV exposure if they exhibited greater than 80% neutralization at the 1:2560 serum dilution.

### NS1 and IgM ELISA Testing for Flaviviruses

Primary DENV screening in patients was performed using a commercial ELISA (Panbio Dengue Early Rapid Test NS1 antigen capture test, Alere, Waltham, MA, USA) following manufacturer’s instructions. For WNV and JEV, a commercial IgM capture ELISA for work with diagnostic specimens in BSL-2 facilities (InBios WNV Detect™ and JEV Detect™, InBios, Seattle, WA, USA) was used following the manufacturer’s instructions.

### RT-PCR

West Nile virus and JEV primers and probes were used to detect the NS2 gene (24). The samples of all WNV and/or JEV IgM-positive samples and mosquito pools were screened. Briefly, RNA was isolated from mosquito pools, human CSF (where received, patients with neurological symptoms only), and serum samples using a commercial virus RNA extraction kit (QIAamp viral RNA kit, Qiagen, Valencia, CA, USA). Mosquito pools were homogenized in PBS to form a 1:10 (v/v) homogenate using sterile pestles in 1.5-ml polypropylene tubes. All samples were stored at −80°C until use.

RNA was reverse transcribed to cDNA (iScript cDNA Synthesis Kit, Bio-Rad, Hercules, CA, USA), and PCR was performed. For the WNV PCR, 1,000 nM of forward and reverse primers and 200 nM of fluorogenic probe were used, and for the JEV PCR, 375 nM of forward and reverse primers and 250 nM of fluorogenic probe were used (Biosearch Technologies, Petaluma, CA, USA) in a 20-µl total reaction volume. Standard cycling conditions consisted of once at 95°C for 2 min followed by 40 cycles of 95°C for 15 s and 60°C for 1 min.

### Plaque Reduction Neutralization Testing (PRNT)

A PRNT was performed to determine the neutralizing capacity of patient serum. Viruses used included the following: WNV (NY99), DENV-1 (TS-SMAN), DENV-2 (NGC), DENV-3 (H87), DENV-4 (H241), and JEV (SA-14-8). All PRNT assays were performed side by side under BSL-3 containment to ensure continuity of data. Four-fold dilutions of heat-inactivated patient serum were mixed with an equal volume of MEM (Corning, Tewksbury, MA, USA) containing ~50–100 plaque-forming units of virus and incubated for 1 h at 37°C. After incubation, this mixture was inoculated onto confluent Vero E6 cells in 12-well plates, which were then incubated for 1 h at 37°C. After incubation, the inoculum was removed; and an overlay of MEM with 0.5% wt/vol methylcellulose, 2.5% FBS, non-essential amino acids, penicillin, streptomycin, and amphotericin B was added to the wells; and the plates were incubated until plaques were visible (3–10 days). For visualization of plaques, the methylcellulose overlay was removed, and cells were stained with a fresh mixture of 50% methanol, 43% ethanol, 7% acetic acid, and 0.1% wt/vol Comassie blue and plaques were counted. The antibody titer was determined as the highest serum dilution that neutralized 50% of the virus inoculum.

### Vector Surveillance

**Figure 2:**
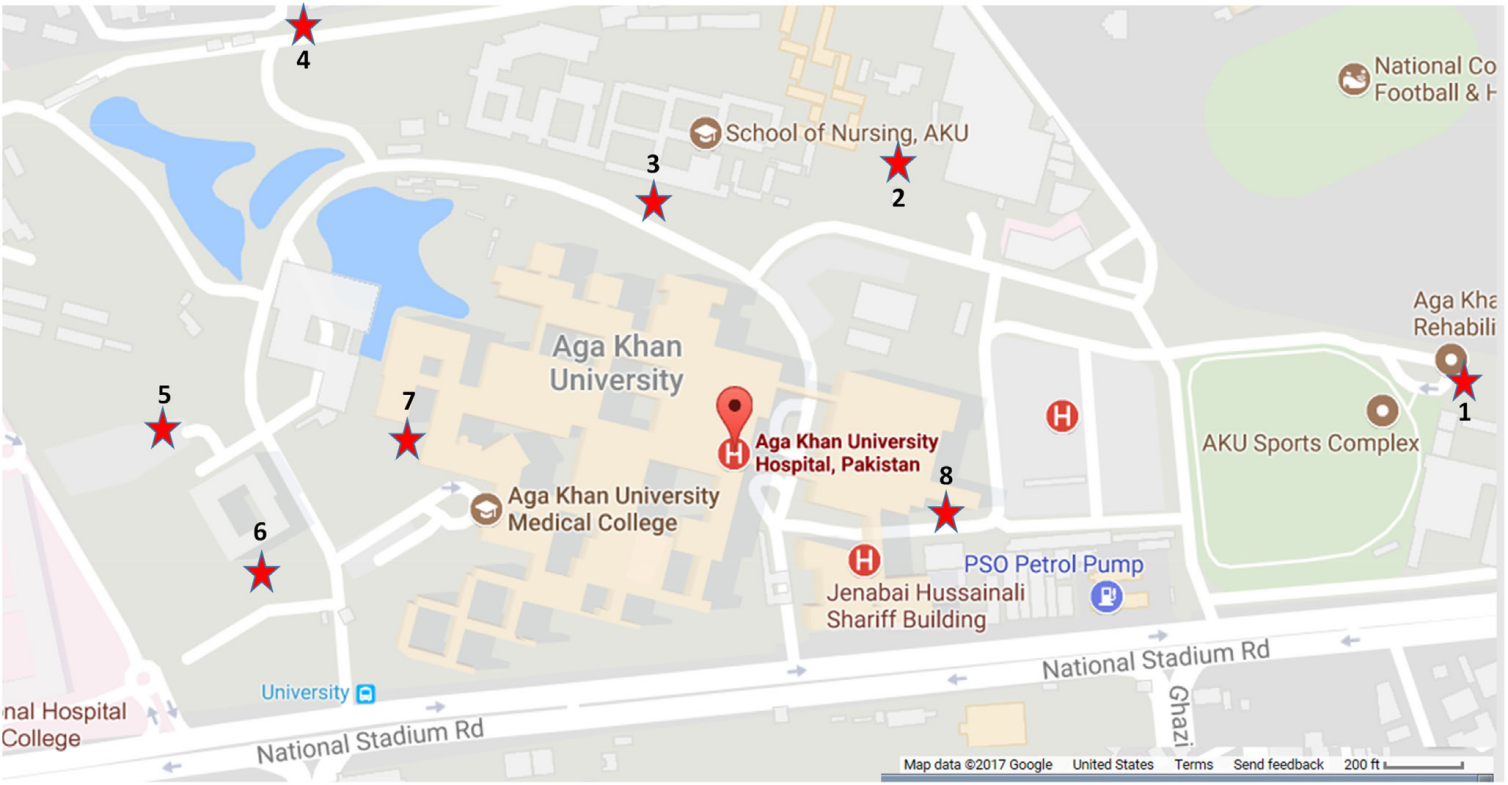
Locations of the mosquito traps used for vector collection, May–November 2015. Sites were selected based on their close vicinity to trees/shrubbery, water, and human activity. The area encompassed 0.34 km^2^ and included high-rise buildings, lakes, water pools, and green patches.

Eight sites (1–8) were selected in Karachi for mosquito trap placement (Figure 2) based on their close vicinity to trees/shrubbery, water, and human activity. The area was spread over 0.34 km^2^ and landscaped with high-rise buildings, lakes, water pools, and intermittent green patches. The average distance between the sites was 200 m.

Three types of mosquito traps were used including the CDC Miniature Light Trap (Model 512), the BG-Sentinel™ (Biogents GmbH, Regensburg, Germany) and the CDC Gravid trap (John W. Hock Company, Gainesville, Florida, model 171). The traps were set per manufacturers’ recommendations. The CDC light trap was hung approximately 2 m above the ground. The BG sentinel trap was baited with the BG-Lure attractant supplied by the manufacturer and placed near an area sheltered from wind and direct sunlight. The Gravid Trap infusion was prepared by adding 3 l of water to dry grass hay. These traps were connected to a 12 V rechargeable battery.

Mosquito surveillance activity began in May 2015 and ended in November 2015. Traps were placed near identified locations for four consecutive nights (Monday to Thursday) from 4:00 p.m. to 9:00 a.m. Each set of traps was placed at one site for 1 week and then rotated to another site. The mosquitoes were collected daily and taken directly to the research laboratory, and mosquitoes were placed on a chilled Petri dish and separated and differentiated according to sex and species. Female mosquitoes species were further separated according to blood and non-blood fed. All identified mosquitoes were quantified and aliquoted and stored at −80°. For each RT-PCR reaction, RNA was extracted from individual pools of 30 mosquitoes. Mosquito grinding and RNA extraction was performed using Qiagen viral RNA extraction kit.

### Environment Monitoring of Temperature and Humidity Charts

The Pakistan Meteorological Department, Karachi head-office graciously shared their daily temperature, humidity, and precipitation monitoring data. Mean temperature, humidity, and precipitation were calculated and recorded. Monthly data compilations were correlated with the number of mosquitoes collected from traps and confirmed human WNV cases.

### Statistical Analysis

Statistical analyses were performed on clinical data using MedCalc version 17.9.7—64-bit. Logistic regression for dichotomous independent variables was performed. Odds ratios were calculated with 95% confidence intervals. Ratios with a *p* less than 0.05 were considered significant. Pearson’s correlation coefficients, and associated *p* values were calculated to identify potential relationships between different variables in the data set.

## Results

### WNV Is a Cause of Febrile Illness in Patients with Undifferentiated Fever in Pakistan

**Figure 3:**
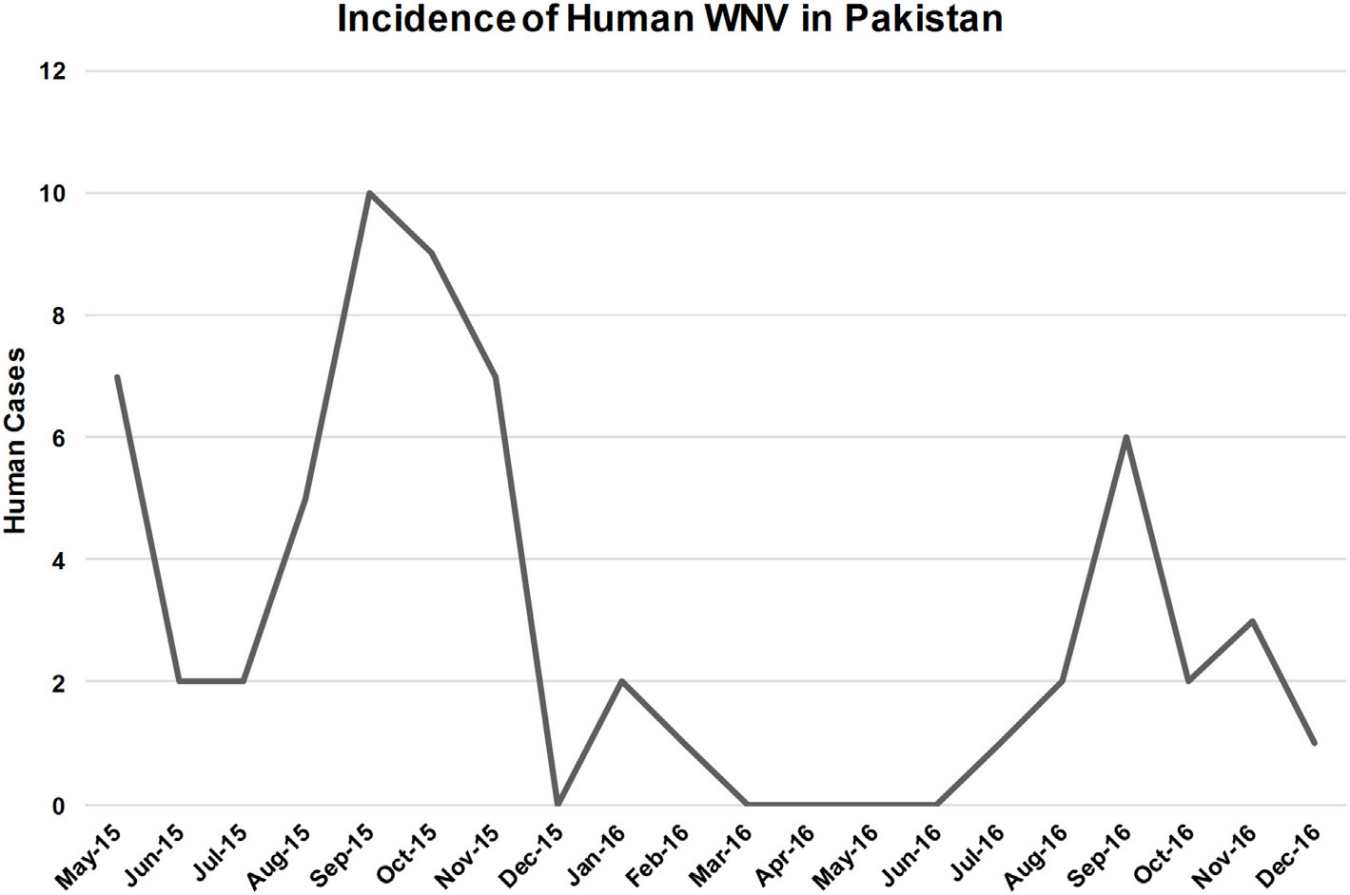
Number of patients with both West Nile virus (WNV)-specific IgM and neutralizing antibodies. Data include patient specimens from all five study sites over a 2-year period.

West Nile virus exposed patients were enrolled from all five sites beginning in May 2015 and continuing through December 2016 (Figure 3). Peaks in human case were observed during September and October, which is consistent with the incidence of WNV reported in other endemic areas like Europe, the United States, and the Mediterranean region (Figure 3). Clinical specimens were confirmed positive based on the CDC guidelines that require detection of virus-specific IgM antibodies with virus-specific neutralizing antibodies in the same or later specimen (23). Of a total of 997 patients enrolled, based on the CDC criteria for clinical manifestations of arboviral disease, 105 patients tested positive for WNV IgM. Twenty-eight (26.6%) of these patients were positive for WNV IgM only (Table S1 in Supplementary Material). Seventy-nine (75.23%) patients exhibited positive IgM for both WNV and JEV (Table S1 in Supplementary Material). Eight (7.5%) of these patients were also positive for DENV NS1 antigen (Table S1 in Supplementary Material).

The 105 samples that were positive for WNV IgM were evaluated for neutralizing antibodies specific for JEV and WNV *via* PRNT. Thirty-six (33.96%) exhibited greater neutralization for WNV than JEV when the minimum neutralization was set at 80% (Table S1 in Supplementary Material). Fifty-nine (55.66%) patients exhibited greater neutralization of WNV than JEV when the minimum neutralization was decreased to 50% (Table S1 in Supplementary Material). In the absence of neutralizing antibodies, patients were classified as probable exposures for the virus for which they possessed the highest IgM activity. Seventeen (16.1%) individuals had greater neutralizing activity for JEV than WNV (Table S1 in Supplementary Material). Twenty-nine (27.36%) patients did not exhibit significant neutralization of either WNV or JEV (Table S1 in Supplementary Material). CSF samples from two patients who presented with signs and symptoms suggestive of viral encephalitis/meningitis were tested by RT-PCR for WNV; however, none of the samples were positive for WNV (data not shown).

### Clinical Features of Human WNV Infection

Of the 105 WNV IgM-positive patients, 61.9% were male (*n* = 65; Table 1). The mean age was 31 years; however, patients from 10 to 86 were enrolled (data not shown). Clinical findings at time of presentation included headache, body ache, and nausea/vomiting and were seen in most patients enrolled. The average duration of fever at time of presentation was 4 (±2) days. Thirty-nine (*n* = 41) presented with signs and symptoms suggestive of central nervous system (CNS) involvement that included altered consciousness, vertigo, seizures, limb weakness, decreased Glasgow Coma scores, and encephalitis (Table 1). For the sake of simplifying data analysis, acute disseminated encephalomyelitis, meningitis, and meningoencephalitis were all classified as encephalitis.

**Table 1:** Incidence of symptoms of suspect and confirmed West Nile virus (WNV)-positive patients compared to WNV-negative patients presenting with clinical features suggestive of arboviral disease.

Symptom	WNV confirmed (*n*)	WNV IgM+ (*n*)	WNV confirmed, OR (95% CI)	WNV suspect, OR (95% CI)
Male	67% (40)	73.5% (64)	0.54 (0.22–1.31)	0.24 (0.05–1.13)
Eye pain	8.4% (5)	9% (8)	1.33 (0.30–5.87)	1.59 (0.18–13.79)
Fever ≥ 38°C	44% (26)	43.6% (38)	1.33 (0.61–2.90)	1.52 (0.55–4.25)
Hemorrhage	18.6% (11)	19.5% (17)	2.09 (0.68–6.44)	4.11 (0.51–33.12)
Rash	11.8% (7)	23.7% (14)	1.24 (0.44–3.52)	0.74 (0.21–2.56)
Nausea	47.4% (28)	59.7% (52)	**0.40 (0.18**–**0.89)**	**0.29 (0.09**–**0.95)**
Headache	42.3% (25)	43.5% (38)	1.04 (0.48–2.28)	1.29 (0.46–3.61)
Myalgia	35% (21)	42.5% (37)	0.66 (0.30–1.45)	0.89 (0.33–2.46)
Arthralgia	15% (9)	13.8% (12)	1.48 (0.46–4.75)	0.78 (0.19–3.13)
Hypertension	10.7% (6)	16% (14)	0.89 (0.29–2.68)	0.54 (0.15–1.93)
Altered mental status	28.3% (17)	24% (21)	**4.25 (1.32**–**13.69)**	**5.45 (0.68**–**43.44)**
Seizures	15% (9)	13.8% (12)	2.58 (0.66–10.14)	2.64 (0.32–21.79)
Limb weakness	5% (3)	6.9% (6)	0.56 (0.12–2.65)	0.52 (0.09–2.93)
Encephalitis	21.7% (13)	22.9% (20)	1.62 (0.62–4.24)	2.75 (0.59–12.88)
Thrombocytopenia	61.7% (37)	71.2% (62)	0.55 (0.23–1.31)	0.36 (0.09–1.34)
Lymphocytosis	33.3% (20)	36.8% (32)	1.17 (0.52–2.65)	0.68 (0.24–1.87)
Reduced Glasgow Coma Scale	10.7% (6)	10.7% (9)	1.96 (0.48–8.06)	423E + 006
Any neurological symptom	40.7% (24)	47.1% (41)	1.65 (0.75–3.62)	**3.58 (1.10**–**11.66)**
Elevated AST	92.6% (25)	61% (63)	0.95 (0.80–1.12)	2.00 (0.49–8.24)
Elevated ALT	68.4% (26)	62.5% (42)	1.2 (0.47–3.34)	1.05 (0.83–1.13)

*Patients with both WNV-specific IgM and neutralizing antibodies were considered confirmed, and patients with only IgM antibodies were considered suspect. Bold values indicate statistical significance for the odds ratio (*p* < 0.05)*.

**Figure 4:**
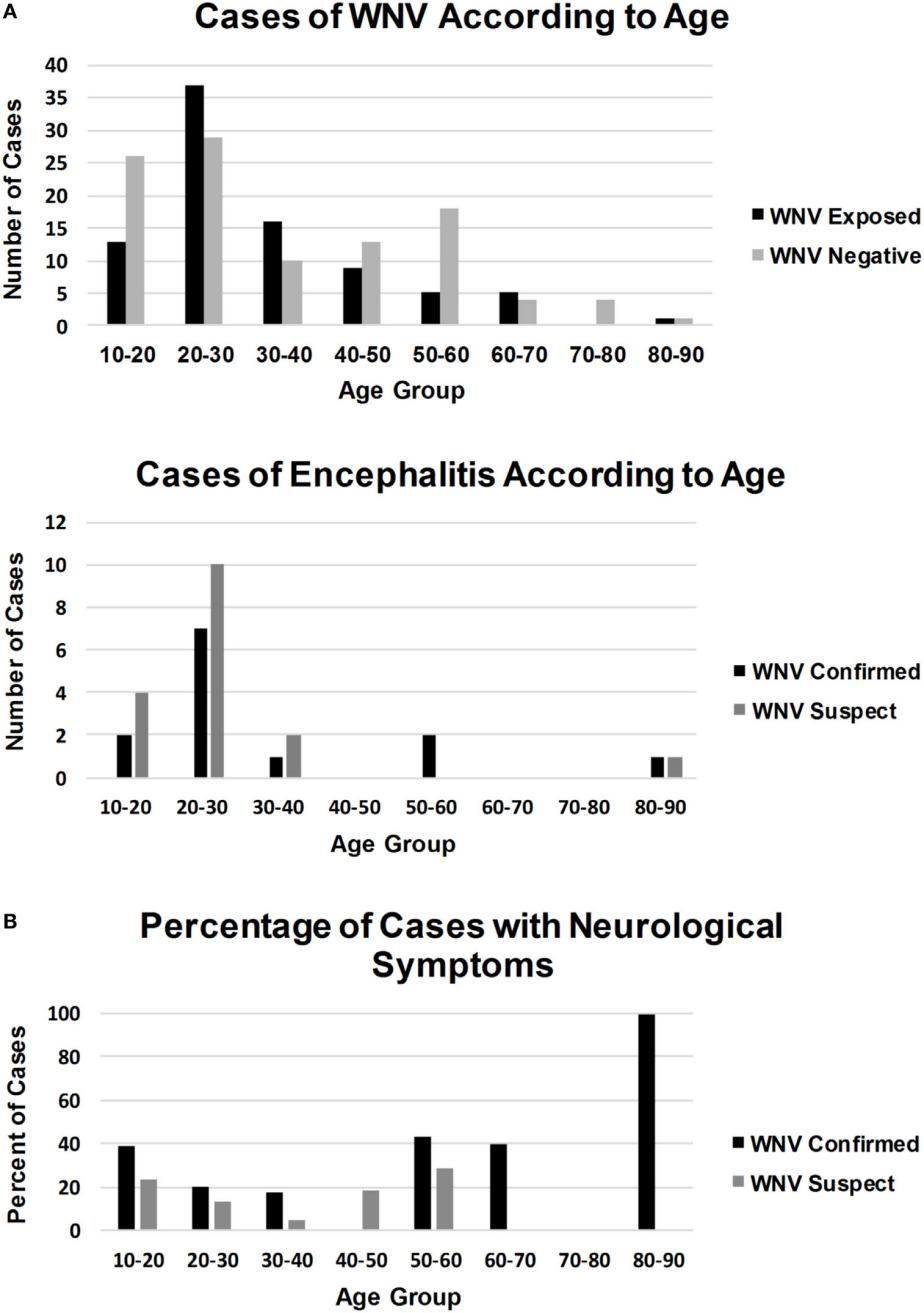

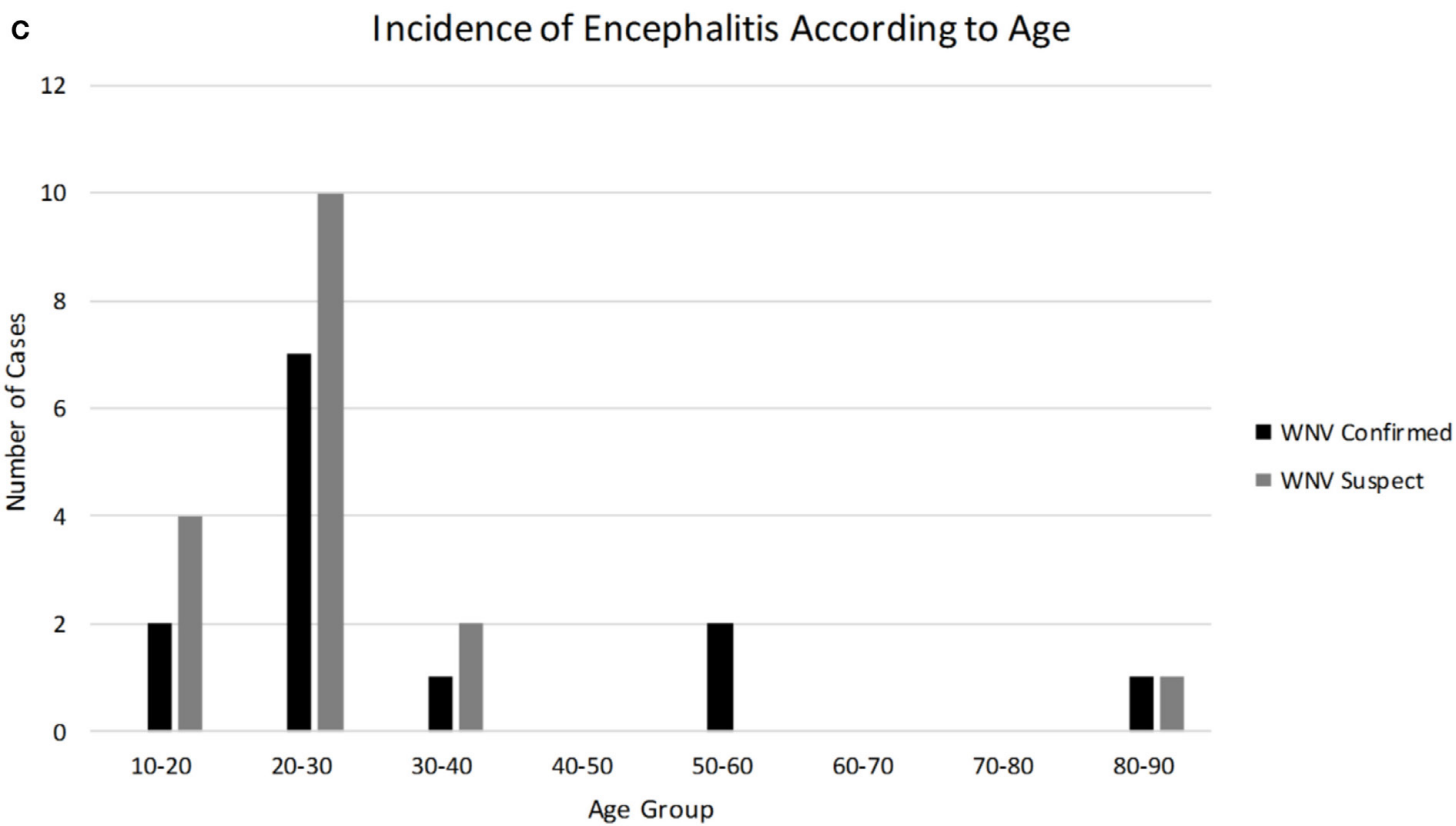
A graphical representation of how WNV infections **(A)**, WNV encephalitis **(B)**, and percentage of cases with neurological symptoms **(C)** occurred in different age groups.

Although males comprised 73.5% of the WNV IgM positive and 67% of the WNV confirmed population, this variable was not significant (Table 1). Clinical symptoms suggestive of WNV neuroinvasive disease were not always indicative of WNV exposure. WNV confirmed individuals were 4.25 times more likely to present with altered mental status (*p* = 0.0058) and twice as likely to have seizures, hemorrhage, and encephalitis (Table 1). Other symptoms of WNV neuroinvasive disease such as stiff neck, vertigo, limb weakness, and reduced Glasgow Coma Scale were not significant predictors of exposure and were just as likely to be observed in individuals negative for WNV exposure (Table 1). Over half of WNV-exposed individuals were younger than 30 years, and 82% were younger than 40 years (Figure 4). Although most patients with encephalitis were in the 20–30 age group, the percentage of encephalitis was highest in the very young or in the 50–60 and 60–70 age groups (Figure 4).

When comparing the symptoms of WNV- and JEV-exposed individuals, several features were unique for each virus. Patients with JEV exposure were 7.16 times more likely to present with GI distress including nausea and vomiting (*p* = 0.0053) and 5.5 times more likely to be male (*p* = 0.0445; Table 2). Persons with JEV exposure were also nearly five times more likely to exhibit thrombocytopenia than WNV-exposed patients (*p* = 0.0438; Table 2). However, altered mental status was not reported for patients with JEV exposure and was 4.25 times more likely to be identified in patients with WNV exposure (*p* = 0.043; Table 2).

**Table 2:** Incidence of symptoms in West Nile virus (WNV) and Japanese encephalitis virus (JEV) confirmed cases in patients presenting with clinical features suggestive of arboviral disease.

Symptom	WNV confirmed (*n*)	JEV confirmed (*n*)	WNV OR (95% CI)	JEV OR (95% CI)
Male	67% (40)	94.4% (16)	0.54 (0.22–1.31)	3.5 (0.75–16.36)
Eye pain	8.4% (5)	5.9% (1)	1.33 (0.30–5.87)	0.73 (0.08–6.29)
Fever ≥ 38°C	44% (26)	35% (6)	1.33 (0.61–2.90)	0.82 (0.28–2.36)
Hemorrhage	18.6% (11)	5.9% (1)	2.09 (0.68–6.44)	0.28 (0.03–2.27)
Rash	11.8% (7)	23.5% (4)	1.24 (0.44–3.52)	1.6 (0.46–5.64)
Nausea	47.4% (28)	94.4% (16)	**0.40 (0.18**–**0.89)**	**7.16 (1.54**–**33.21)**
Headache	42.3% (25)	41% (7)	1.04 (0.48–2.28)	0.72 (0.24–2.11)
Myalgia	35% (21)	41% (7)	0.66 (0.30–1.45)	1.06 (0.37–3.05)
Arthralgia	15% (9)	11.8% (2)	1.48 (0.46–4.75)	0.84 (0.17–4.16)
Hypertension	10.7% (6)	17.6% (3)	0.89 (0.29–2.68)	1.36 (0.34–5.44)
Altered mental status	28.3% (17)	0% (0)	**4.25 (1.32**–**13.69)**	7.68E–010
Seizures	15% (9)	0% (0)	2.58 (0.66–10.14)	2.37E–009
Limb weakness	5% (3)	11.8% (2)	0.56 (0.12–2.65)	0.85 (0.09–7.58)
Encephalitis	21.7% (13)	5.9% (1)	1.62 (0.62–4.24)	0.19 (0.02–1.5)
Thrombocytopenia	61.7% (37)	94.4% (16)	0.55 (0.23–1.31)	3.94 (0.85–18.42)
Lymphocytosis	33.3% (20)	41% (7)	1.17 (0.52–2.65)	1.4 (0.48–4.06)
Reduced Glasgow Coma Scale	33.3% (20)	0% (0)	1.65 (0.75–3.62)	2.40E–009
Any neurological symptom	40.7% (24)	41% (7)	0.95 (0.80–1.12)	**0.13 (0.03**–**0.62)**
Elevated AST	10.7% (6)	71.4% (10)	1.2 (0.47–3.34)	0.57 (0.14–2.31)
Elevated ALT	40.7% (24)	73.3% (11)	0.79 (0.21–2.99)	0.95 (0.74–1.22)
Dengue/WNV co-infection and neurological symptoms	14.8% (4)		2.16 (0.4–11.74)	
Prior Dengue viruses exposure WNV neuroinvasive disease	11% (3)		**0.18 (0.03**–**0.99)**	

*Patients with both virus-specific IgM and neutralizing antibodies were considered confirmed. For cross-reactive patients, exposure was confirmed for the virus with the greatest Plaque Reduction Neutralization Testing titer. Bold values indicate statistical significance (*p* < 0.05)*.

Unfortunately, immune status ratios (ISRs) for the WNV and JEV IgM kits did not correlate with neutralization *via* PRNT (*p* = 0.76 WNV, *p* = 0.12 JEV; Table S1 in Supplementary Material). Many WNV and JEV ISR values indicated positive exposure, but the samples had little to no neutralizing activity (Table S1 in Supplementary Material). Moreover, several WNV ISR values indicated WNV exposure, but when assayed *via* PRNT, these patients had greater neutralizing activity for JEV than WNV (Table S1 in Supplementary Material). In the absence of neutralizing antibodies, specimens were classified as suspect/probable exposure based on whichever virus exhibited the greatest ISR values.

### WNV and JEV IgM Responses Are Highly Cross-Reactive

A subset of 28 patients with JEV and WNV IgM antibodies were evaluated for neutralization of DENV. These patients were all positive for WNV, DENV, and JEV IgM. PRNTs were performed for these patients against DENV 1-4, WNV, and JEV (Table S1 in Supplementary Material). All but three patients had at least 80% neutralizing activity against at least one DENV serotype at antibody titers of 1:40 (Table S1 in Supplementary Material). Two of the three non-DENV neutralizing patients did not exhibit neutralizing activity against WNV or JEV despite having encephalitis and limb weakness and positive IgM antibodies (Table S1 in Supplementary Material). The remaining patient had neutralizing antibodies to WNV only and presented with altered mental status and meningitis. Sixteen of the 28 patients exhibited serological profiles indicative of prior DENV infections with DENV neutralization of at least 80% at the 1:2560 serum dilution (Table S1 in Supplementary Material). Three of these patients exhibited symptoms of neuroinvasive disease, but having prior exposure to DENV was not a contributing factor (*p* = 0.0238; Table S1 in Supplementary Material). Here again, DENV ISR values did not correlate with neutralizing activity (*p* = 0.2931 DENV1, *p* = 0.3157 DENV2, *p* = 0.4099 DENV3, *p* = 0.6602 DENV4) nor were the ISR values dependent on whether the patient had a primary or secondary DENV exposure (*p* = 0.9861; Table S1 in Supplementary Material).

### DENV/WNV Co-infections Are Associated with Neurological Symptoms

Dengue virus NS1 antigen was detected in eight patients with WNV-specific IgM and neutralizing antibodies. Four patients exhibited symptoms of neuroinvasive disease and were twice as likely to exhibit neurological symptoms than patients without DENV co-infection (Table S1 in Supplementary Material).

### Vector Abundance Correlates with Temperature and Precipitation

**Figure 5:**
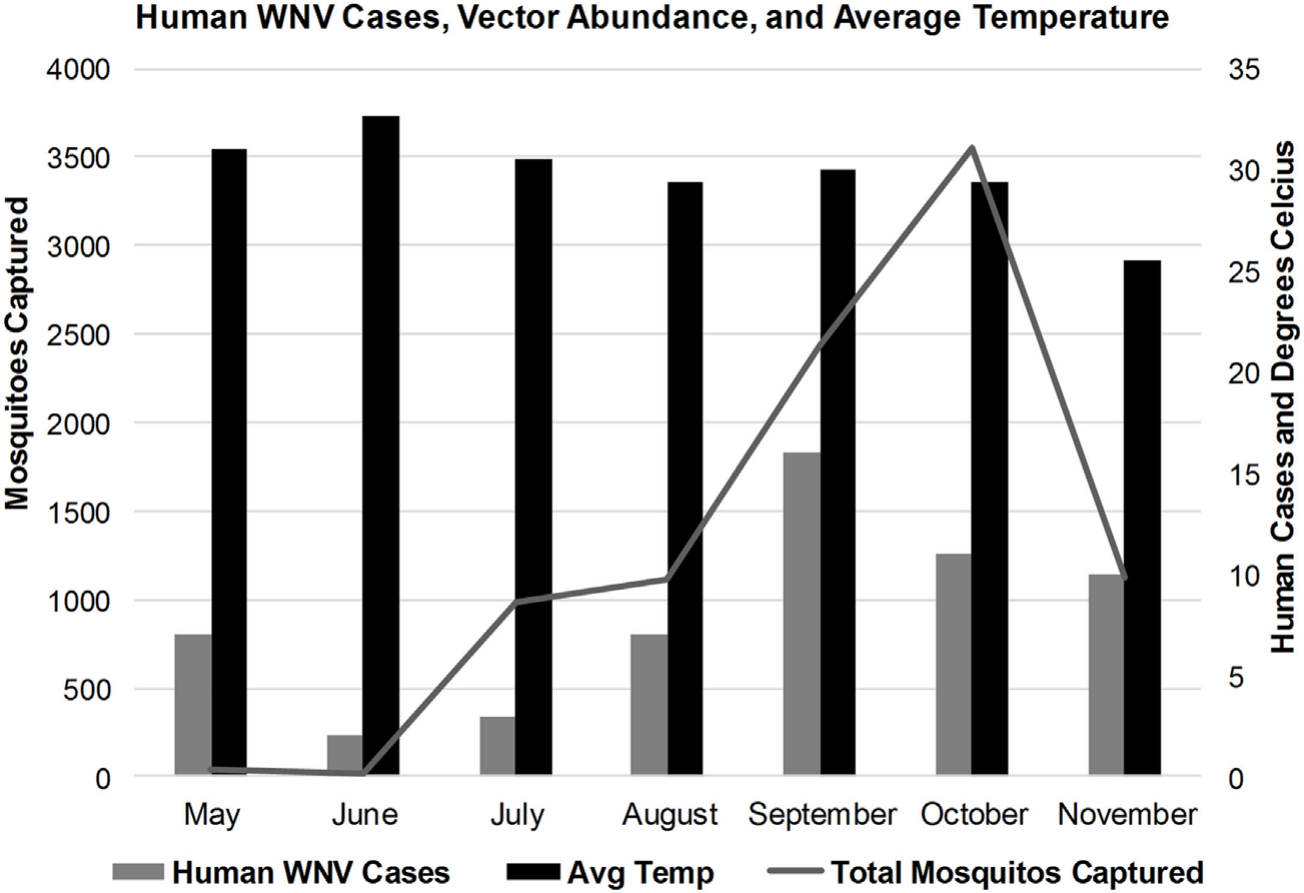
Correlation of human West Nile virus cases, vector abundance, and average monthly temperatures in Karachi, Pakistan.

Temperature recordings for Karachi during 2015 showed an increase in temperatures in May through October with average temperatures between 31 and 30°C. Peak temperatures occurred in June (average = 33C°; Figure 5). During this period, Karachi experienced the worst heat wave on record for June with temperatures reaching 43°C. During this time, significantly fewer mosquitoes were collected (Figure 5). The average humidity showed an upward trend starting in May that continued through October. During November, Karachi experienced a fall in average temperatures by 3–4° and a drop in relative humidity by 20% compared to the preceding months (Figure 5).

The average number of mosquitoes collected in the traps correlated with peak temperatures of the city when the heat waves were subtracted from the analysis (*r* = 0.8067) (Figure 4). A total of 8,931 mosquitoes were collected during the study period. The majority of mosquitoes belonged to the *Culex* species (Table 3). 47.5% of the total number of mosquitoes were male *Culex*, while female *Culex* represented 51.8% of the total (Table 3). *Anopheles* (male 0.27%; female 0.34%) and *Aedes* (male 0.03%; female 0.097%) species accounted for very small percentages of the total (Table 3). Mosquito numbers were lowest in the months of May and June (*n* = 52); however, numbers increased significantly during July (*n* = 984) and August (*n* = 1,110) with a peak in September (*n* = 2,434) and October (*n* = 3,553; Figure 5). A sharp decline in mosquito numbers was recorded in November (*n* = 1,125; Figure 5). It was during the month of September that WNV was detected in a pool of 30 *Culex* mosquitoes that were trapped at location 8 (Figure 2), which is near the entrance to the offices for the Aga Khan University Hospital.

**Table 3:** Numbers, species, and sex of mosquitoes caught in three types of trap.

Trap	Genus	Male	Female	Total
CDC gravid	*Culex*	2,466	3,365	5,831
BG sentinel	*Culex*	1,566	1,315	2,881
CDC light	*Culex*	62	91	153
CDC gravid	*Anopheles*	9	18	27
BG sentinel	*Anopheles*	14	13	27
CDC light	*Anopheles*	0	0	0
CDC gravid	*Aedes aegypti/Aedes albopictus*	1	6	7
BG sentinel	*A. aegypti/A. albopictus*	2	3	5
CDC light	*A. aegypti/A. albopictus*	0	0	0

Of the three types of traps used in this study, the CDC Gravid Trap was the most successful in capturing *Culex spp*., catching 65.7% of all *Culex spp*. (Table 3). This was twice as effective as the BG Sentinel and 38 times more effective than the CDC light Trap (Table 3). For *Anopheles* and *Aedes* species, the CDC Gravid Trap and the BG-Sentinel performed the same (Table 3). The CDC Light Trap did not capture any *Anopheles* or *Aedes* species and overall was the least successful, contributing only 2% to the total number of mosquitoes caught (Table 3).

## Discussion

Diagnosis and appropriate treatment of arboviral disease is problematic in areas where these pathogens co-circulate. Overlapping clinical presentation combined with serological cross-reactivity render definitive diagnosis expensive and cumbersome. Furthermore, secondary infections with these types of viruses can exacerbate clinical symptoms and complicate interpretation of diagnostic tests. Laboratory confirmation remains a major challenge, and RT-PCR is not very useful in diagnosis of WNV or JEV as viremia occurs 3–4 days before the onset of clinical symptoms. Given that DENV, JEV, and WNV are flaviviruses, there is serological cross-reactivity and absolute confirmation requires the most cumbersome and highly skilled plaque reduction assay. This type of infrastructure is lacking in many labs and medical facilities in Pakistan.

The data herein emphasize that viral IgM is not indicative of exposure. Patients positive for JEV IgM were usually positive for WNV IgM and *vice versa*. Even more troubling was that these cross-reactive samples were also invariably positive for DENV IgM (Table S1 in Supplementary Material). It was also impossible to achieve a diagnosis based on the IgM activity alone as ISR values were often very close in value and could not be directly compared between viruses due to test composition and result parameters. Analysis of the PRNT data showed no correlation between IgM activity with neutralizing activity. There was also no correlation between IgM positivity and PRNT positivity at any serum dilution. As most of the epidemiological data being generated from lower- and middle-income countries is based on IgM diagnostics, without further confirmatory testing or collection of convalescent serum, this raises questions as to the true prevalence for these viruses.

Similar diagnostic issues were present for neutralizing antibodies as most patients exhibited some level of neutralization for WNV, JEV, and DENV 1–4. Similar neutralizing activity has been reported for DENV and Zika virus, WNV and Murray Valley encephalitis virus, JEV with DENV 1–4, and Yellow fever virus with WNV, DENV, and tick-borne encephalitis virus (25–28). We also noted a large background of prior DENV exposure indicated *via* highly neutralizing PRNT titers, which mirrors what has been reported in other studies examining flaviviral cross-reactivity (26, 28).

When following the CDC/WHO diagnostic algorithms for arboviral disease, cross-reactivity devalues their use if IgM ELISA and PRNT are used as diagnostic tools. As stated by Lee et al. “Prior to the introduction of Zika to the Americas, PRNT was considered the gold standard of serological assays” (26). Cross-reactive samples used to be diagnosed as “flavivirus exposed” or some other generic classification. Since effective treatments are not available for most flavivirus infections, a definitive diagnosis was not viewed as significant as it is growing to be. Emerging data documenting debilitating sequelae including arthralgia and neurological issues, the development of neurodegenerative disorders, and horrific congenital consequences emphasize the need for rapid and accurate diagnostics to drive decisions and reassure patients and health care providers (26, 29–33).

Dengue viruses are endemic in Pakistan, and as such, many patients enrolled in this study were diagnosed as having DENV fever by physicians although neurological symptoms were present. It is not uncommon for DENV to co-circulate WNV and/or JEV, which can lead to misdiagnosis as most of the symptoms overlap. These viruses present as a flu-like illness characterized by fever, myalgia, headache, gastrointestinal disturbance, and maculopapular rash. Up to 1% of WNV-/JEV-infected individuals may show neuroinvasive disease (34, 35). Clinical features like headache, altered mental status, GI distress, limb weakness, and motor neuron disease pattern with flaccid paralysis are clues to the diagnosis of CNS infection with WNV or JEV (34, 35). Over a third of WNV-confirmed patients here had clinical features consistent with neuroinvasive disease. WNV-exposed patients exhibited unique symptoms when compared to JEV. WNV-infected individuals were much more likely to exhibit altered mental status, reduced Glasgow Coma scores, encephalitis, and seizure activity, whereas persons with JEV infection were more likely to suffer from nausea, thrombocytopenia, and be male.

The data show that average summer temperatures correlated with human WNV incidence. Mosquito numbers and human WNV infections and precipitation were also directly correlated. It was during this time that WNV was detected in a pool of *Culex spp., via* RT-PCR, which suggests that active WNV transmission was occurring. These findings are concordant with similar reports in Europe (35, 36). The city of Karachi is an ideal urban environment for *Culex* spp. propagation and transmission. The city has vectors, hosts, and an abundance of standing water with sewage or other organic material that enhance larval propagation (37–39). The low numbers of *Aedes* and *Anopheles* spp. collected was most likely due to our trapping methods. No trapping occurred during the daytime when *Aedes* mosquitoes are active and CO_2_ or other baits for attracting *Anopheles* were not used (40). Further surveillance of larger geographical areas along with larval and pupal demographic surveys are required to assess overall picture of the various mosquito species in Karachi.

A major limitation of this study was that only patient serum was tested *via* RT-PCR for the presence of virus. Since the inception of this study, research in molecular diagnostics of clinical specimens has determined that whole blood and urine are more useful for detecting WNV in human patients (41). Another limitation of the study is that vector surveillance was conducted in a small geographic region of Karachi (0.34 km^2^), and sentinel animals were not used. Despite these limitations, our results can be generalized as most of Karachi has similar features in terms of mosquito propagation sites, avian fauna, and susceptible human population.

Detection of vectors and humans infected with WNV is suggestive of a competent enzootic cycle. The climatic and sanitation conditions in Karachi are supportive of active propagation and dissemination of vectors and consequent risk of an epidemic outbreak of this virus. Active disease surveillance and preventive strategies are not available in Pakistan but are urgently needed at regional and national levels to prevent such from happening in timely manner. Moreover, there is as a great need for the standardization of diagnostic assays and procedures, more accurate diagnostic assays, and renovated diagnostic algorithms for Pakistan and other countries where WNV co-circulates with other flaviviruses. While development of surveillance networks, diagnostic assays, and standardized procedures can be overwhelming for developing countries, it is rapidly becoming apparent that the long-term costs to public health are greater in the face of flavivirus disease. Given the high seroprevalence of flaviviruses, reinstitution of DEWS or similar program should be seriously considered for Pakistan.

Regardless, the findings of this study are an alert for physicians to suspect WNV infection in patients with altered mental status and other neurological symptoms in a dengue-like illness in patients inhabiting or traveling to and from Pakistan, especially during the late summer and autumn months. WNV-associated neuroinvasive disease is an important differential diagnosis for other neurological diseases such as poliovirus currently circulating in Pakistan.

## Ethics Statement

Patients were enrolled under informed consent procedures that were reviewed and approved by the Ethics Review Committee, Aga Khan University (3183-PAT-ERC-14) and the Institutional Review Board, University of Florida (201500908). All enrolled subjects gave written informed consent in accordance with the Declaration of Helsinki.

## Author Contributions

The following authors contributed to this manuscript in the following ways: contribution to the conception and design of the work: MTL, EK, KLB, JAL; acquisition, analysis and interpretation of data: MTL, EK, DP, KLB, KI, SA, ZYK, ZA, AA, JF, FM; drafting, editing, revising and approving drafts: MTL, KLB, EK, KI, DP, JF, FM, JAL. MTL, EK, and KLB all agree to be accountable for all aspects of the work.

## Conflict of Interest Statement

The authors declare that the research was conducted in the absence of any commercial or financial relationships that could be construed as a potential conflict of interest.
