# Corrigendum: Human West Nile Virus Disease Outbreak in Pakistan, 2015–2016

**DOI:** 10.3389/fpubh.2018.00384

**Published:** 2019-01-29

**Authors:** Erum Khan, Kelli L. Barr, Joveria Qais Farooqi, Dhani Prakoso, Alizeh Abbas, Zain Yar Khan, Shanze Ashi, Kehkashan Imtiaz, Z. Aziz, Faisal Malik, John A. Lednicky, Maureen T. Long

**Affiliations:** ^1^Department of Pathology and Laboratory Medicine, Aga Khan University, Karachi, Pakistan; ^2^Department of Comparative Diagnostic and Population Medicine, College of Veterinary Medicine, University of Florida, Gainesville, FL, United States; ^3^Department of Environmental and Global Health, Emerging Pathogens Institute, University of Florida, Gainesville, FL, United States

**Keywords:** West Nile virus, Dengue virus, Japanese encephalitis virus, encephalitis, arboviral disease

An author name was incorrectly spelled as “Alizae Abbas.” The correct spelling is “Alizeh Abbas.” Additionally, the author name “Zain Y. Khan” was incorrectly spelled. The correct spelling is “Zain Yar Khan.”

The authors apologize for this error and state that this does not change the scientific conclusions of the article in any way. The original article has been updated.

